# Free-living greylag geese adjust their heart rates and body core temperatures to season and reproductive context

**DOI:** 10.1038/s41598-018-20655-z

**Published:** 2018-02-01

**Authors:** Claudia A. F. Wascher, Kurt Kotrschal, Walter Arnold

**Affiliations:** 1 Core facility Konrad Lorenz Forschungsstelle for Behahviour and Cognition, University of Vienna, Fischerau 11, A-4645 Grünau im Almtal, Austria; 2 Department of Biology, Anglia Ruskin University, East Road, Cambridge, CB1 1PT United Kingdom; 3 Department of Behavioural Biology, University of Vienna, Althanstrasse 14, A-1090 Vienna, Austria; 4 Research Institute of Wildlife Ecology, University of Veterinary Medicine, Vienna, Savoyenstraße 1, A-1160 Vienna, Austria

## Abstract

Animals adaptively regulate their metabolic rate and hence energy expenditure over the annual cycle to cope with energetic challenges. We studied energy management in greylag geese. In all geese, profound seasonal changes of heart rate (*f*_H_) and body temperature (T_b_) showed peaks in summer and troughs during winter, and also daily modulation of *f*_H_ and T_b_. Daily mean *f*_H_ was on average 22% lower at the winter trough than at the summer peak, whereas daily mean T_b_ at the winter trough was only about 1 °C below the summer peak. Daily means of T_b_ together with those of air temperature and day length were the most important predictors of daily mean *f*_H_, which was further modulated by precipitation, reproductive state, and, to a minor degree, social rank. Peaks of *f*_H_ and T_b_ occurred earlier in incubating females compared to males. Leading goslings increased daily mean *f*_H_. Our results suggest that in greylag geese, pronounced changes of *f*_H_ over the year are caused by photoperiod-induced changes of endogenous heat production. Similar to large non-hibernating mammals, tolerance of lower T_b_ during winter seems the major factor permitting this. On top of these major seasonal changes, *f*_H_ and T_b_ are elevated in incubating females.

## Introduction

In endothermic animals, low ambient temperatures increase energy requirements to maintain high core body temperature (T_b_)^1^. This is particularly problematic for herbivores which often simultaneously face severe food shortages. The result is a direct survival risk that may affect fitness in following seasons^2^. Daily torpor or hibernation are strategies for down-regulating metabolic rate (MR) and T_b_, thereby adjusting energy management when faced with food shortage and/or low ambient temperatures (for review see^3,4^). An evident cost to both strategies is constrained manoeuvrability and behavioural reactivity. Therefore, daily torpor and hibernation are typically associated with the availability of shelters. However, large non-hibernating mammals, such as red deer (*Cervus elaphus*), Przewalski’s horse (*Equus ferus przewalskii*), Alpine ibex (*Capra ibex*), or Shetland pony (*Equus caballus*) also show substantial reduction of winter energy expenditure. These mammals do so by employing similar physiological mechanisms compared to hibernators or daily heterotherms, *i*.*e*. via a reduction of endogenous heat production and tolerance of lower T_b,_ particularly in peripheral parts of the body^5–11^. Nevertheless, the reduction of core T_b_ is only minor in these species and manoeuverability is certainly not impaired to the extent present in hibernators and daily heterotherms.

In birds, reduced basal metabolic rate (BMR)^12^, and associated facultative hypothermia occurs in many species as a reaction to food shortage or low ambient temperatures; one species, the common poorwill (*Phalaenoptilus nuttallii*) is even known to hibernate (reviewed in^4,13^). However, most studies of seasonal metabolic adjustments in birds found that BMR is up-regulated during winter (reviewed in^14^). It has been suggested that seasonal changes in standard metabolic rate (SMR)^12^ depend on body size, *i*.*e*. increasing during winter in small birds, but decreasing in large birds (>200 g^15^). Hitherto, the empirical evidence for this remains equivocal^14^ and the picture is even more obscured by seasonal migration, a strategy to escape harsh winter conditions.

Besides extreme temperatures and food shortage, reproduction and moult pose major energetic challenges^16^. Birds are known to increase energy expenditure during different phases of reproduction, *e*.*g*. egg production, incubation, nestling provisioning^17–19^. Two competing hypotheses about energy allocation in seasonally breeding birds have been suggested: The ‘increased demand hypothesis’ predicts that the total energy demand is greater during reproduction than during winter and hence, a seasonal peak in MR is expected during the reproductive phase. Crucially, changes in energy expenditure over the annual cycle strongly depend, according to this hypothesis, on seasonal changes in activity patterns and the energy content of food^20,21^. However, the geese in our study were *ad libitum* fed also during winter. Therefore, we can rule out the availability and energy content of the food to be the driver for changes in MR. In line with the ‘increase demand hypothesis’ we would expect little T_b_ variation across the year, but an increase in MR during the reproductive period.

In contrast, the ‘reallocation hypothesis’ predicts relative little variation in T_b_ and MR during the annual cycle. During winter, birds are expected to have higher energy expenditure due to increased thermoregulatory needs. This additional energy expenditure is, according to the ‘reallocation hypothesis’, reallocated to reproductive activity during the breeding season, because birds breed when ambient temperatures are moderate^22^. Hitherto, little empirical support has been produced for either hypothesis^21,23^. Based on what is known about the winter physiology of non-hibernating mammals, we here suggest ‘winter hypometabolism’ as a third hypothesis for explaining seasonal changes of MR. For large birds, such as geese, this hypothesis predicts a decrease in MR during winter mainly due to reduced endogenous heat production and a tolerance of lower T_b_^8,24^, particularly during the nocturnal rest phase^5,7^. Further the winter hypometabolism hypothesis predicts higher MR and T_b_ during summer independent of reproduction, *i*.*e*. also occurring in not reproducing birds. Therefore, in contrast to previous studies which mostly considered changes in activity patterns and energy content of food as drivers for changes in MR, we here predict that a change of endogenous heat production, accompanied by a down-regulation of T_b_, is the most important modulator of MR.

In the present study, we test these hypotheses via data loggers implanted into 25 individuals of a flock of free-living, non-migrating, individually marked greylag geese (*Anser anser*). The loggers stored 2-minute means of *f*_H_, an acknowledged proxy of field metabolic rate (FMR)^25–28^, and abdominal T_b_ for a maximum of 18 months. FMR is measured in free-living animals under natural conditions, in contrast to BMR and SMR, which measure MR under standardized conditions, *e*.*g*. during rest, in a specific environment regarding temperature.

## Results

**Figure 1:**
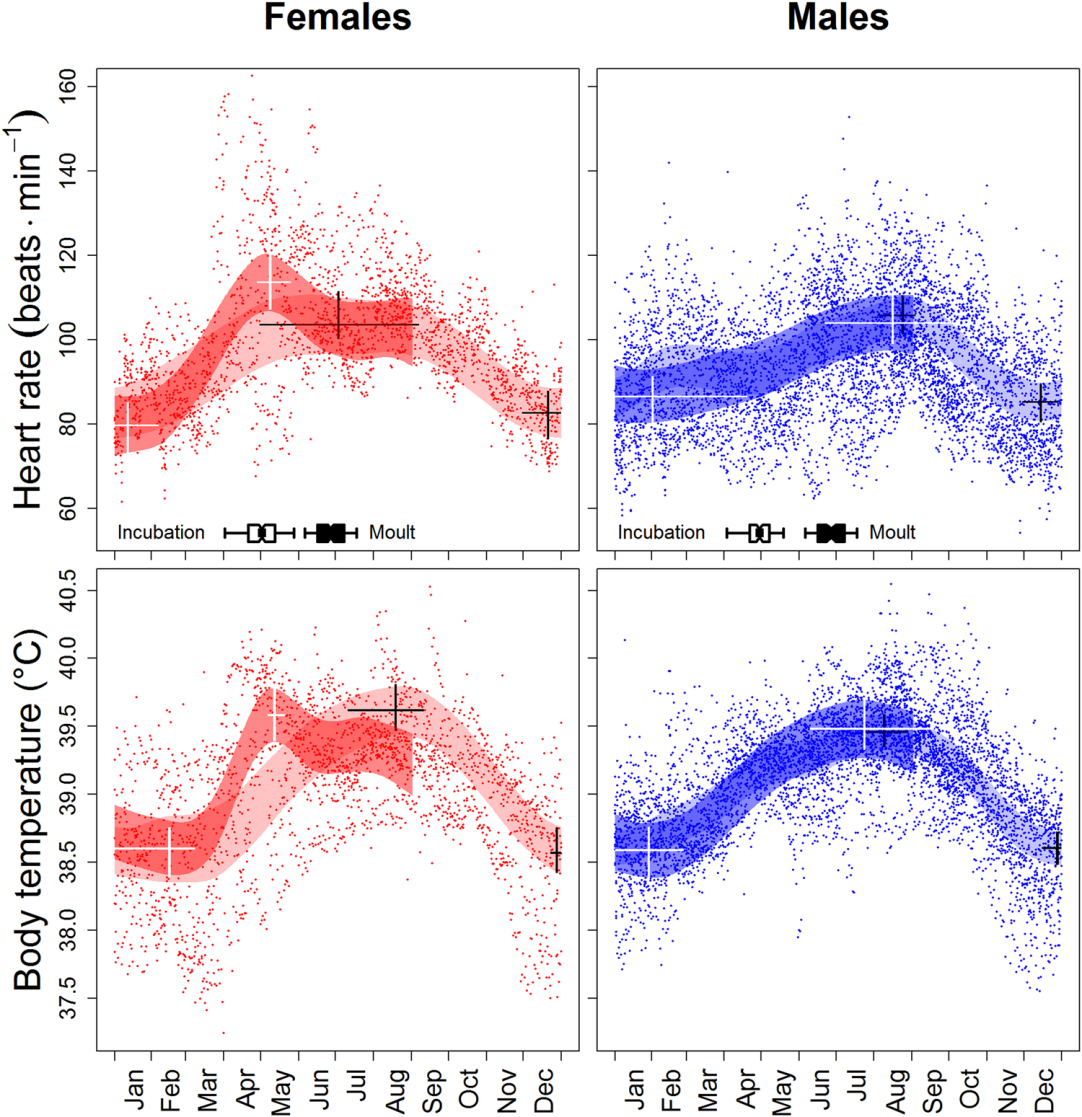
Annual course of heart rates and core body temperatures of female (red) and male geese (blue). Plotted values are daily individual means. Periods of incubation and moult are shown as box-plots at the bottom of the upper graphs. Shaded areas indicate 95% confidence belts of the overall mean courses determined by spline fitting (see Methods for details). Horizontal bars within belts indicate 95% confidence limits of the location of local peaks and troughs, vertical bars 95% confidence limits of the height of these peaks and troughs, respectively. If horizontal bars extend from December to January only the part in either of these months is plotted. Dark shaded and white bars: reproductive animals; light shaded and black bars: non-reproductive animals.

Daily mean heart rate (*f*_H_) and body core temperature (T_b_) both varied profoundly over the year, with peaks during summer and troughs during winter. The magnitude of these peaks and troughs was similar in reproductive and non-reproductive females and males (Fig. 1). Compared to the summer peaks, mean *f*_H_ was about 22% lower at the winter troughs. Conversely, despite virtually identical seasonal patterns, average T_b_ at the winter troughs was only about 1 °C below the summer peaks (Fig. 1). Troughs of *f*_H_ and T_b_ occurred during the period of December to March. Peak *f*_H_ and T_b_ occurred between July and September for most birds, with the exception of reproductive females, which had highest *f*_H_ and T_b_ during the incubation period in April/May (Fig. 1). The period of moult coincided largely with the period of highest *f*_H_ for males and non-reproductive females (Fig. 1), although this was not a statistically significant effect (p = 0.636, Table 1).

**Table 1:** Results of mixed linear modelling of power transformed daily means of heart rate.

	Females (df for age = 5, all others = 2248)				Males (df for age = 15, all others = 5727)				difference between sexes (df for age = 20, all others = 7975)	
	Partial coefficient	Standardized coefficient	t-value	p-value	Partial coefficient	Standardized coefficient	t-value	p-value	t-value	p-value
Intercept	0.44594		3.814	<0.001	−0.42111		−5.455	<0.001	4.030	<0.001
Daily mean body temperature	0.06222	0.728	21.171	<0.001	0.08578	0.719	44.754	<0.001	6.921	<0.001
Daily mean air temperature	−0.00266	−0.500	−11.705	<0.001	−0.00195	−0.320	−17.271	<0.001	3.253	0.001
Photoperiod	0.01363	0.738	5.726	<0.001	0.00511	0.251	4.032	<0.001	−3.280	0.001
Precipitation	−0.00002	−0.038	−3.363	<0.001	−0.00003	−0.043	−8.399	<0.001	−0.613	0.540
Age	−0.00368	−0.235	−1.622	0.166	−0.00432	−0.194	−1.214	0.243	−0.030	0.976
% won interactions	−0.00042	−0.209	−2.123	0.034	0.00019	0.104	1.550	0.121	2.540	0.011
Incubating	−0.03873		−4.907	<0.001	−0.02775		−3.115	0.002	0.911	0.362
Leading goslings	0.03105		3.330	0.001	−0.02278		−2.404	0.016	−4.207	<0.001
Moulting	0.00283		0.473	0.636	0.00122		0.366	0.714	−0.270	0.787

Continuous predictors are listed in the order of effect size according to standardized coefficients, categorical predictors in the order of effect size according to t-values. Reported p-values are the type I error probabilities of the effect of each predictor after adjusting for the effects of all other predictors in the statistical model.

Among all variables and factors tested for independent associations with daily mean *f*_H_, daily mean T_b_ was by far the strongest predictor of *f*_H_. T_b_ correlated positively with *f*_H_ in both sexes with a slightly stronger effect in females (Table 1). In females the second strongest predictor of *f*_H_ was photoperiod and the magnitude of this effect was much greater than in males (Table 1). Mean air temperature and the amount of precipitation per day were negatively related to daily mean *f*_H_ in both sexes, but again with stronger effects of air temperature observed for females (Table 1). During incubation females showed comparatively lower *f*_H_ after correcting for their higher T_b_ during this period the same effect was found in the attending male partner (Table 1). Leading goslings after hatching in both sexes was associated with higher *f*_H_ (Table 1). Age had no discernible effect on *f*_H_ in either sex. The percentage of won interactions during aggressive interactions had only weak effects. Interestingly, this indicator of rank in the flock was positively associated with *f*_H_ in males but negatively in females (Table 1).

**Figure 2:**
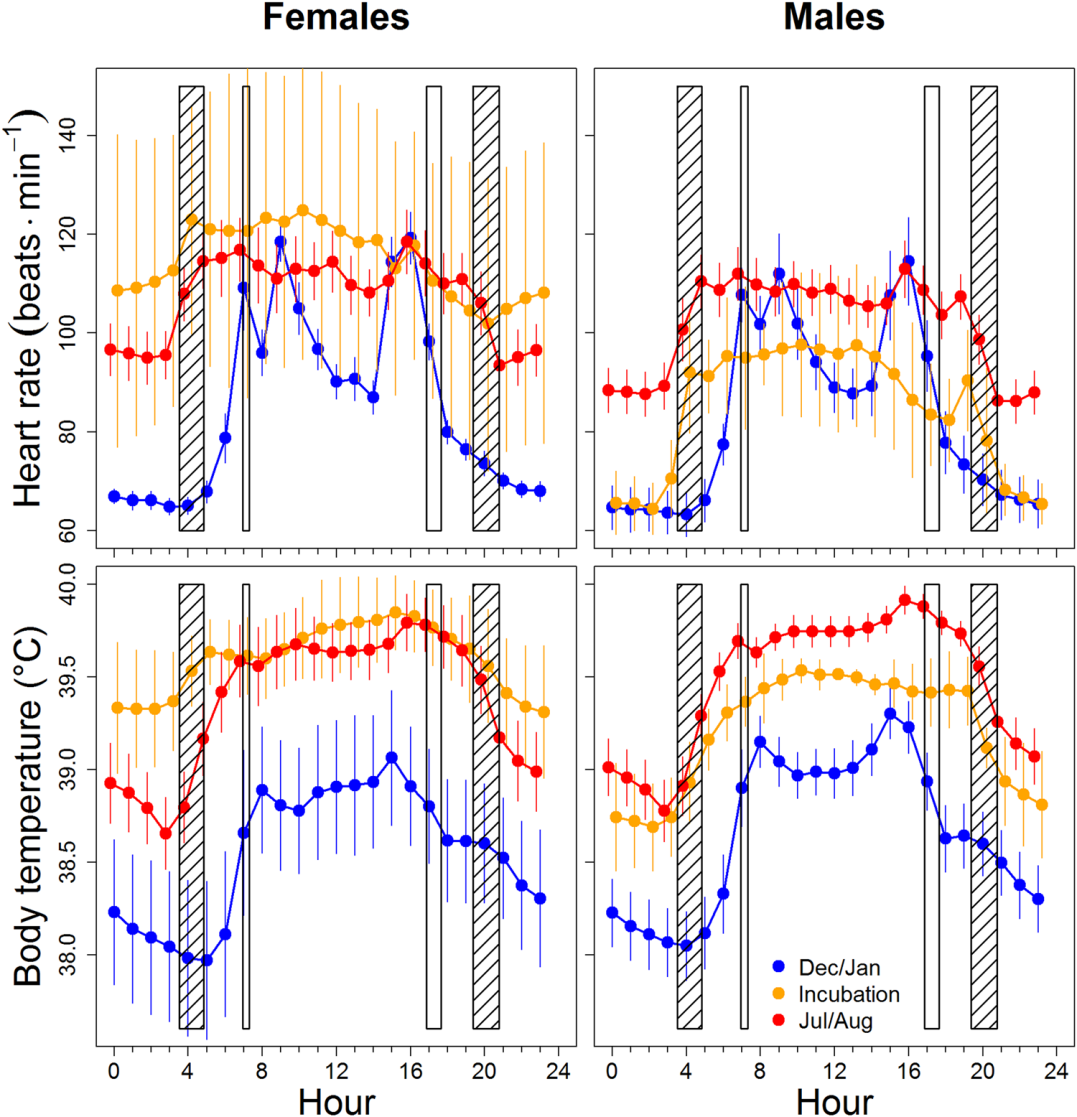
Daily course of heart rates and core body temperatures of female and male geese during December and January (blue), July and August (red), and of incubating females and their mates (orange). Plotted are hourly means of heart rate and body temperature. Error bars represent 95% confidence intervals of these means and reflect variation between individuals. Vertical bars indicate the range of onset and end of civil twilight in the morning and evening, respectively, at the roosting site Almsee. Open bars: December/January); hatched bars: July/August.

The correspondence between *f*_H_ and T_b_ on an annual scale (Fig. 1) was also evident on a daily scale (Fig. 2). The pattern found in hourly means of the *f*_H_ and T_b_ during summer (July/August) and winter days (December/January) resembled the typical courses of *f*_H_ and T_b_ of diurnal animals, *i*.*e*. lower *f*_H_ and T_b_ during the night. However, T_b_ was significantly lower over the day during December/January as compared to July/August. The same difference was present in *f*_H_ except during early morning and late afternoon hours in December/January when pronounced peaks of *f*_H_ occurred (Fig. 2). Both, *f*_H_ and T_b_ were highest in incubating females with apparently minor differences between day and night, in contrast to the patterns found in their mates over the same time period (Fig. 2).

## Discussion

The changes in heart rate (*f*_H_) recorded over the annual cycle indicate a reduction of field metabolic rate (FMR) during winter in the observed population of greylag geese. Among all variables and factors tested for independent associations with daily mean *f*_H_, daily mean T_b_ was by far the strongest predictor of *f*_H_. Such a correlated winter drop in T_b_ and *f*_H_ contradicts both, the ‘increased demand hypothesis’ and the ‘reallocation hypothesis’; but supports our novel hypothesis of ‘winter hypometabolism’. Among the potential predictors of *f*_H_ tested, T_b_ had by far the strongest effect on *f*_H_ in both sexes (Table 1). This result suggests that the reduction of *f*_H_ is mainly due to a decreased endogenous heat production and simultaneous down-regulation of T_b_. The closely associated patterns of *f*_H_ and T_b_ evident over the day and over the year support this view. Changes in locomotor activity may also have contributed to changes of *f*_H_ and T_b_^10,11,29^. As we have no quantitative simultaneous measurements of activity along with the measurement of *f*_H_ and T_b_, we could not include this factor into our analysis. Still, activity may significantly contribute to seasonal changes of *f*_H_, although the contribution was presumably minor as compared to that of T_b_, as has been found in other species^5,7–9,24^. Furthermore, the higher morning and afternoon peaks of *f*_H_ during January and December, as compared to July and August, most likely resulted from increased activity triggered by our feeding schedule. The food provided during the winter months was particularly attractive to the geese and likely caused increased locomotor activity during the two daily feeding periods^29^. Together with heat increment of feeding this may have constrained the otherwise more continuous metabolic depression during winter.

A profound decrease of FMR during winter, however, was found despite our *ad libitum* feeding, indicating that photoperiod rather than food availability is the major cue governing the annual cycle of *f*_H_ and T_b_^30–32^. After correcting for other factors, photoperiod indeed showed the second-strongest influence on *f*_H_ in females and the third-strongest influence in males. Independent of these effects, unfavourable weather conditions, *i*.*e*. low air temperatures and considerable precipitation also increased *f*_H_^33,34^. However, high precipitation increased mean *f*_H_ similarly in both sexes, whereas low air temperatures had a greater effect on female than male *f*_H_. This sex difference may be due to lower body mass of females^35,36^, rendering them more prone to heat loss.

We observed a modulation of endogenous heat production not only as a result of photoperiod-induced seasonal acclimatization and unfavourable weather conditions, but also in response to other energetically costly situations, such as reproduction. For example, we found a significantly earlier annual peak of *f*_H_ in incubating females as compared to non-incubating females. The higher energy expenditure of incubating females in contrast to non-incubating birds apparently results from the need to maintain high endogenous heat production and T_b_ even through the night in order to keep eggs warm. Furthermore, the energetic cost of, again photoperiod-induced, follicle development may have also contributed to the spring peak of *f*_H_ in reproducing females^37^. On a first glance, these results seem to support the ‘increased demand hypothesis’ and parallel previous findings of an increased energy expenditure during reproduction in female birds, for example in great tits *Parus major*^38^, Arabian babblers *Turdoides squamiceps*^39^, or macaroni penguins *Eudyptes chrysolophus*^40^. However, it has to be pointed out that in contrast to females, the incubation period in males was characterized by low *f*_H_. This presumably reflects the low energy expenditure due to decreased locomotor activity, which is not only true for the incubating female, but also for the attending partner, as in greylag geese, the male pair-partner tends to stay close to the incubating female during the entire incubation period. Also, contrary to what would be predicted by the ‘increased demand hypothesis’, T_b_ during incubation actually increased in females. This again supports the ‘hypometabolism hypothesis’ predicting that MR is more strongly associated with T_b_, rather than locomotor activity and caloric intake.

In geese, only females incubate the eggs and brood the offspring. It has been argued that male investment into reproduction mostly consists of vigilance and agonistic behaviour against conspecific competitors, which ensures paternity on side of the male^41^, and access to resources such as food and nesting sites on the female side^42^. However, the energetic cost of such behaviour was apparently low, because we did not find a spring peak of *f*_H_ in males but a seasonal course similar to that of non-reproductive females (Fig. 1). Moreover, leading goslings was associated with low *f*_H_ in males. Therefore, there is obviously no ‘increased demand’ for males during the period of reproduction, in contrast to females.

Further, in all our geese, we found a late summer high of *f*_H_ (Fig. 1). This peak was not associated with moult as in Barnacle geese (*Branta leucopsis*)^43^. The period of moult roughly coincided with the parental period in the birds studied, and had no discernible effect on *f*_H_ independent of other factors tested. Barnacle geese, however, moult later than greylag geese and the coincidence of the annual peak of MR with moult in Barnacle geese may therefore be spurious. In greylag geese, other factors than reproduction or moult must be responsible for the late summer peak of *f*_H_. We suggest pre-migration fattening^44^ as a possible explanation for this, i.e. an ‘increased demand’, but later in the year as originally proposed by this hypothesis. Increased frequency of social interactions in the newly formed summer flock^45^ may further contribute to the late summer peak of *f*_H_. Finally, we found a sex-dependent effect of dominance rank, *i*.*e*. percentage of aggressive interactions won, onto *f*_H_: The higher-ranking a male the higher *f*_H_. Although this effect was minor, it supports previous results indicating sex-specific social investment in geese^46^.

In conclusion, our results indicate that the pronounced changes of *f*_H_ during the year are mainly caused by photoperiod-induced changes of endogenous heat production. Tolerance of lower T_b_ during winter is a major factor in this. Hence, our results support our ‘hypometabolism hypothesis’. Comparison with males and non-reproducing females demonstrates that in reproducing females T_b_ and FMR is further elevated during incubation, superimposed on baseline seasonal changes.

## Methods

### Study population

A non-migratory flock of greylag geese was introduced in the Almtal (Upper Austria) by late Konrad Lorenz in 1973. The geese are unrestrained and roam the valley between the Konrad Lorenz Forschungsstelle (47°48′N, 13°56′E) and a lake approximately 10 km to the South (Almsee; 47°44′N 13°57′E), where they roost at night. At the time of our study the flock consisted of approximately 170 individuals, marked with coloured leg bands for identification. The flock was supplemented with pellets and grain twice daily (0800–0900; 1500–1700), creating a condition of nearly *ad libitum* food provisioning year round. Both hand-raised and goose-raised flock members were habituated to the close presence of humans and neither show avoidance if approached up to 1 m distance, nor did they significantly change *f*_H_ when familiar humans approached^47^.

### Heart rate and body temperature measurements

Twenty-five individuals (8 females/17 males) were fitted with a battery-powered electronic package (60 × 30 × 11 mm, ~60 g; for further details see^36^), fully implanted into the abdominal cavity. Packages contained a VHF-transmitter, battery, antenna, a temperature sensor, memory, and were encapsulated in a physiologically inert medical-grade silicone rubber. Two electrode plates (surgical steel, approximately 8 mm in diameter and 1 mm thick), fixed towards the lower rib cage in the vicinity of the heart, detected the QRS complex of the electrocardiogram and were connected to the electronic package with approximately 10 cm long coiled silicone rubber-insulated wire of multistranded stainless steel, fitted into silicone rubber tubing to form an elastic and flexible lead. Heartbeats were counted by a microcontroller and average values for consecutive 2-minute intervals were stored on board. Accuracy of *f*_H_ measurement was ± 2 beats per minute (bpm). Output of the temperature sensor was also stored on board at the end of each consecutive 2- minute interval. Each sensor was calibrated before implantation in the range of 35–41 °C. We were able to re-calibrate 6 devices after surgical removal upon termination of the experiment. We could not detect a significant drift in any of these devices.

### Ethics

All procedures performed in this study involving animals were in accordance with Austrian law and the institutional guidelines of the University of Veterinary Medicine, Vienna. Implantations were performed by an experienced team of veterinarians^48^ under the animal experiment license issued by the Austrian Ministry of Science GZ68.210/41-BrGT/2003).

### Weather data

Daily mean air temperature and per day-accumulated precipitation was provided by a weather station at the Almsee, run by the ‘Hyrdographischer Dienst’ of Upper Austria.

### Photoperiod

For each day of the year we calculated onset and end of civil twilight at the roosting site Almsee according to http://lexikon.astronomie.info/zeitgleichung/ to determine minutes of daylight and night and to calculate the length of photoperiod.

### Observations

Presence of each individual goose and pair-bond status was monitored every other day in a standard way by experienced observers^49^. Additionally, during the reproductive season, nests were monitored and information on numbers of eggs, onset of incubation, hatching date and numbers of hatchlings was collected. For each family, the number of young (goslings) was recorded every day until fledging of the young, at approximately three months of age. When focal individuals had dependent offspring, this parental period of the year was defined as ‘leading goslings’^49^. Lengths of the incubation periods were determined from these observations for each breeding female and its partner. We considered a female and its pair-partner as reproductive during a year when the female was incubating, regardless of whether goslings hatched and survived. Data from reproductive individuals were not available for the months September to December (Fig. 1). A couple of weeks after hatching of the young, geese moult their wing feathers and are unable to fly for a few weeks^50^. Duration of the moulting period was determined for each individual of the flock by observations every other day.

The percentage of won aggressive encounters of each individual was evaluated during summer, fall, and mating season by observing social interactions before and during the daily feedings on five days per period (for details see^51^). The overall percentage was used as an indicator of an individual’s dominance rank in the flock in a given period.

### Data analyses

For the analyses presented here, we used *f*_H_ and T_b_ data downloaded from the surgically removed electronic packages. We discarded the first days of measurements after implantation of a device because of potential aftermaths of surgery on *f*_H_ and T_b_. Periods before surgical removal of the transmitters, containing obvious outlier values, which are likely due to dwindling battery power, were also discarded. Raw *f*_H_ data were purged with a moving average filter to remove biologically implausible outlier values. All statistical analyses were preformed using R 3.3.0^52^.

We tested continuous and categorical variables potentially associated with seasonal changes of *f*_H_ in linear mixed models (R-package ‘nlme’^53^), with daily mean *f*_H_ calculated for each individual as response variable. We explicitly included photoperiod as a predictor of *f*_H_ to correct for the influence of seasonal differences in day length and hence the period of daily activity and associated *f*_H_ and T_b_ in these diurnal birds. By including 2-way interactions of all predictors with sex we further tested whether effects differed between females and males. For these analyses, we power-transformed daily mean *f*_H_ in order to achieve a normal distribution of residuals.

For visualizing seasonal changes of *f*_H_ and T_b_, we calculated general additive mixed models (R-package ‘mcgv’^54^ with spline fits to day of the year as predictor variable of *f*_H_ and T_b_ for reproductive and non-reproductive females and males, and 95% confidence intervals (CI) of these fits. CI of the timing and height of local trough and peak *f*_H_ and T_b_, respectively, were determined by bootstrapping from respective distributions of troughs and peaks produced by simulating 10000 replicates of model coefficient vectors from the posterior using ‘mvrnorm’ from R-package MASS^55^.

In all models, we accounted for repeated measures by including individual identity as a random intercept factor and corrected for temporal correlation detected in the residual error term of models by including an auto-regressive correlation structure (‘corARMA’ from R-package ‘nlme’).
